# Analysis of the Correlation Between Helper T‐Lymphocyte Subsets and Atrial Fibrillation: An Observational and Mendelian Randomization Study

**DOI:** 10.1155/cdr/4440768

**Published:** 2026-08-04

**Authors:** Jiahui Li, Yafen Yang, Hongxuan Fan, Zhuolin Huang, Yalin Yuan, Rui Bai, Xin Wang, Bin Liang

**Affiliations:** ^1^ Department of Cardiology, The Second Hospital of Shanxi Medical University, Taiyuan, Shanxi, China, sxmu.edu.cn; ^2^ Graduate School, Shanxi Medical University, Taiyuan, China, sxmu.edu.cn; ^3^ Department of Cardiology, Beijing Tsinghua Changgung Hospital, School of Clinical Medicine, Tsinghua University, Beijing, China, tsinghua.edu.cn; ^4^ Department of Rheumatology Immunology, The Second Hospital of Shanxi Medical University, Taiyuan, Shanxi, China, sxmu.edu.cn

**Keywords:** atrial fibrillation, case–control study, helper T-lymphocyte subsets, immune system, Mendelian randomization

## Abstract

**Background:**

Atrial fibrillation (AF) is the predominant arrhythmia. Growing evidence indicates that immunological diseases may play a role in its development. This study combined observational and genetic analyses to investigate how T‐lymphocyte subsets and immunomodulatory drug targets influenced AF risk.

**Methods and Results:**

This study retrospectively analyzed T lymphocyte subsets in 302 AF patients and 183 healthy controls. After balancing covariates via 1:1 propensity score matching, multivariate regression revealed AF patients had significantly higher absolute counts of Th1 and Th17 cells, and higher Th1/Th2 and Th17/Treg ratios (*p* < 0.05). A subsequent two‐sample Mendelian randomization (MR) analysis, which assessed 731 immune traits and included drug‐target MR, established causal relationships. It showed that increased counts of CD4^+^CD8dim T cells (OR = 1.03, *p* = 0.002) and IgD^−^CD38dim B cells (OR = 1.06, *p* = 0.0003) elevated AF risk. Furthermore, IL‐6R‐mediated CRP elevation increased AF risk (OR = 1.33, *p* = 5.18E‐7), whereas higher IL‐18 gene expression was protective (OR = 0.95, *p* = 0.036).

**Conclusion:**

Immune dysfunction, especially T lymphocyte subsets, is a risk factor for the development of AF. The IL‐6R signaling pathway may be a potential target for AF prevention, whereas the use of IL‐18 inhibitors may increase the risk of AF.

## 1. Introduction

### 1.1. Epidemiology and Pathological Mechanisms of Atrial Fibrillation (AF)

AF, the most prevalent cardiac arrhythmia, affected 33.5 million people globally in 2010 [1]. AF leads to consequences such as heart failure, thromboembolism, and dementia, as well as reduced quality of life and higher mortality [2]. AF is classified into paroxysmal and persistent types. Paroxysmal AF typically terminates spontaneously within 48 h, whereas persistent AF lasts more than 7 days and may require intervention for termination [3].

AF is a complex arrhythmia whose mechanisms are not well understood. Age is the biggest risk factor [4], followed by gender, smoking, alcohol, body mass index (BMI), hypertension, and heart failure [5]. The primary pathophysiology involves atrial remodeling, including structural, electrophysiological, and molecular alterations [6]. Inflammatory mediators affect calcium homeostasis, modulate connexins, and activate fibroblasts to modify atrial structure [7]. Determining the underlying causes of AF remains challenging and the outcomes of current treatments are suboptimal. Therefore, there is an urgent need for innovative treatments due to the increasing incidence of AF.

### 1.2. AF and Immune Dysfunction

Recently, accumulating evidence has suggested that immune dysregulation plays a pivotal role in the onset and progression of AF. As part of the immune system, the lymphatic system and its cells may contribute to the immunopathogenesis of AF [8]. The four most studied CD4^+^T cell subsets are Th1, Th2, Th17, and Treg cells. A recent cohort study suggested that circulating immune cells are associated with new‐onset AF [9].

### 1.3. Epidemiological and Mendelian Randomization (MR) Studies Were Used to Explore the Relationship Between AF and Immune Disorders

Currently, no international study has investigated the association between absolute lymphocyte subgroup counts and AF risk. Therefore, we investigated the role of absolute counts of lymphocyte subpopulations in the development of AF using a case–control study (CCS) and MR studies. A CCS is a method of epidemiological research that focuses on inferring associations between past exposures to a disease by comparing cases with the disease to controls who do not have the disease. MR methods [10] use genetic variation to infer causal relationships between exposures and outcomes. In the present study, we first applied a two‐sample MR approach to assess causality between immune cells and AF risk. We then used drug‐targeted MR to investigate the effects of immunomodulatory drugs on AF.

## 2. Materials and Methods

### 2.1. CCS

#### 2.1.1. Study Design and Target Population

**Figure 1:**
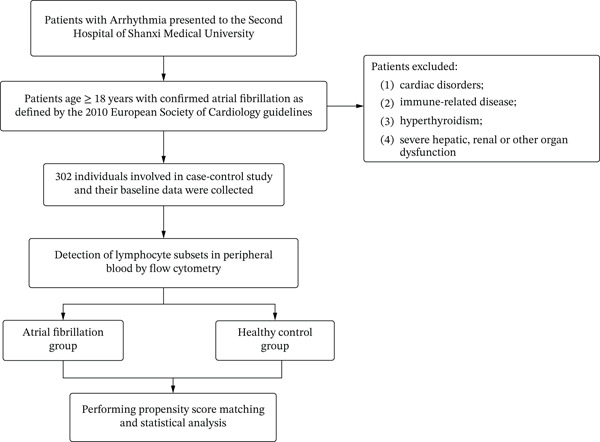
Case–control study flow chart.

The retrospective CCS enrolled 302 AF patients from the Department of Cardiovascular Medicine and 183 healthy controls from the Health Screening Department between June 1, 2020, and November 30, 2023. Among them, AF patients were selected strictly according to the inclusion and exclusion criteria. Inclusion criteria: ① age ≥ 18 years, ② meeting the diagnostic criteria for AF as defined by the 2010 European Society of Cardiology guidelines, ③ completion of standardized lymphocyte subset and T cell subset testing during hospitalization, and ④ complete case data with no missing essential information. Exclusion criteria: ① cardiac system diseases: congenital heart disease, rheumatic heart disease, severe valvular disease and/or mechanical heart valves, and cardiomyopathy; ② immune‐related diseases: malignant tumors, autoimmune diseases, severe infections, and acute and chronic inflammation of various systems; ③ hyperthyroidism; and ④ severe hepatic, renal or other organ dysfunction. Data collected for the CCS included age, sex, height, weight, BMI, history of smoking, drinking, hypertension, diabetes mellitus, and coronary artery disease (CHD). Ancillary investigations comprised lymphocyte subpopulation analysis, T‐lymphocyte subpopulation analysis, routine blood test, liver function, kidney function, thyroid function, lipids, electrolytes, cardiac enzymes, cardiopulmonary tetrograms, coagulation studies, and cardiac ultrasonography, including left ventricular ejection fraction (LVEF) and left atrial size. Markers of immune dysfunction included the Th1/Th2 ratio and Th17/Treg ratio, whereas clinical characteristics included the CHA2DS2‐VASc score, HAS‐BLED score, and presence of thrombosis. Peripheral blood lymphocyte subsets and CD4^+^T cell subsets were detected by flow cytometry. The study was approved by the ethics committee of the Second Hospital of Shanxi Medical University (ID [2022]: YX No. 093), and written informed consent was not required due to the retrospective study design. The flow of the CCS is shown in Figure 1.

#### 2.1.2. Statistical Methods

Propensity score matching analysis (PSM) reduced confounders in CCSs. When including healthy controls and AF patients, we selected age and sex, calculated propensity scores and constructed a well‐matched cohort using the PSM method through SPSS software to ensure the sample was balanced for these variables. For continuous variable data description, the Shapiro–Wilk test was used to assess normal distribution. Mean and standard deviation (SD) were used to describe normally distributed data, whereas the median and interquartile range (IQR) were employed for nonnormally distributed data. Categorical variables were described by frequencies and rates (%) and compared using the Chi‐square test. Depending on data distribution, several statistical approaches were utilised to compare data discrepancies between the two groups. The unpaired *t*‐test was used to compare normally distributed data, whereas the Wilcoxon rank sum test or Mann–Whitney test was used for nonnormally distributed data. To account for multiple hypothesis testing, *p* values were adjusted for the false discovery rate (FDR). Adjusted *p* values are denoted as *p*_FDR_. A significance threshold of FDR < 0.05 was adopted. Univariate and multivariate logistic regression identified independent predictors, and we assessed multicollinearity via variance inflation factor (VIF < 5).

### 2.2. MR Study

#### 2.2.1. Study Design and Target Population

A two‐sample MR analysis assessed causality between 731 immune cell traits and AF risk. From the corresponding genome‐wide association studies (GWAS), 731 tools for immune cell characteristics [11] and AF [12] were selected. For the exposure tool variables, we used a GWAS dataset of 3757 Sardinians analyzed by flow cytometry (Table S1). The genetic dataset for AF was derived from a large meta‐analysis, which comprised 1,030,836 European‐origin subjects who were categorized into 60,620 AF cases and 970,216 controls (Table S2). The overall design is shown in Figure S1.

#### 2.2.2. Selection of instrumental variables (IVs)

IVs were selected at a genome‐wide significance threshold (*p* <1 × 10^−5^) [13]. Linkage disequilibrium (LD) clumping (*R* ^2^ < 0.1, window = 500 kb) was applied to ensure independence of the single nucleotide polymorphisms (SNPs) [14]. To mitigate weak instrument bias, we calculated the proportion of phenotypic variance explained (PVE) and the *F*‐statistic for each IV, and classified those with *F* > 10 as strong IVs for subsequent analyses. Furthermore, to avoid pleiotropic bias, we used PhenoScanner V2 [15] to identify and remove IVs associated (*p* <1 × 10^−5^) with known AF risk factors such as BMI, smoking status, hypertension, CHD, chronic renal failure, and diabetes mellitus (Table S3).

#### 2.2.3. Statistical Methods

All analyses were performed using R version 4.3.2. The primary causal estimates were derived using the inverse variance weighted (IVW) method [16]. The weighted median (WM) [17], simple mode, and weighted mode were used as complementary methods to assess the consistency of the IVW results. Sensitivity analyses included Cochran′s *Q* test for heterogeneity, the MR‐PRESSO method [18] to detec and correct outliers, and the MR‐Egger intercept test [19] to assess horizontal pleiotropy. Causal estimates were adjusted for multiple testing, with a significance threshold of *p* < 0.1 [20], and leave‐one‐out (LOO) analysis [21] was conducted to determine whether any single SNP biased the causal estimates.

### 2.3. Drug‐Targeted MR Study

#### 2.3.1. Study Design and Target Population

Multiple immunomodulatory agents were considered in this study, including colchicine, interleukin (IL) inhibitors, tumor necrosis factor (TNF) inhibitors, T cell activation inhibitors, B cell antagonists, Janus kinase (JAK) inhibitors, and interferon (IFN) inhibitors. Figure S2 summarizes the two research parts of this study. (1) Study 1(GWAS‐based MR): We employed a two‐sample MR framework to evaluate the causal effect of C‐reactive protein (CRP)—a validated inflammatory and cardiovascular risk marker [22] on AF, using genetic proxies for immunomodulatory drug targets. (2) Study 2 (eQTL‐based MR): We applied summary data–based MR (SMR) using expression quantitative trait loci (cis‐eQTL) data of drug target genes to assess their potential causal roles in AF.

CRP genetic data were sourced from the largest available GWAS meta‐analysis of European ancestry (*N* = 575,531) [23] (Table S2). In Study 1, rheumatoid arthritis (RA) was included as a positive control outcome [24] (Table S2).

#### 2.3.2. Selection of IVs

Target genes for immunomodulatory medications were identified using the DrugBank database [25] (Table S4), and their genomic coordinates were annotated via Genecard [26]. SNPs associated with CRP levels were extracted from target gene regions and their neighboring 250 kb windows. SNPs in low LD (*R* ^2^ < 0.2) were retained under a Bonferroni‐corrected *p* value threshold (*P* < 0.05/*n*, where *n* is the cumulative number of SNPs extracted from the gene). For the eQTL analysis, cis‐eQTLs for the identified target genes were obtained from the eQTLGen Consortium (https://www.eqtlgen.org/) (Table S2). We selected significant, common (MAF > 1%) cis‐eQTLs (*p* < 5.0 × 10^−8^) located within a 1‐Mb window of the gene transcription start site as IVs.

#### 2.3.3. Statistical Methods

##### 2.3.3.1. GWAS‐Based MR Analysis

We applied the same MR analysis framework as described in section 1.2.3, utilizing IVW, WM, simple mode, and weighted mode methods, alongside sensitivity analyses for heterogeneity, pleiotropy, and outlier influence. A positive control analysis with RA as the outcome was performed to validate the genetic instruments.

##### 2.3.3.2. eQTL‐based MR analysis

The primary analysis utilized the SMR method [27] to test associations between gene expression and AF. Alleles were coordinated and analysed using SMR software, version 1.3.1. The heterogeneity in dependent instruments (HEIDI) test [27] was applied to distinguish causal associations from linkage (*p* < 0.01 indicated rejection of a causal relationship) [28]. To evaluate potential horizontal pleiotropy, SMR analysis was extended to cis‐eQTLs of all genes within a 1 Mb window of the primary target.

## 3. Results

### 3.1. Case‐control study

#### 3.1.1. PSM Between Healthy Controls and Patients With AF

**Figure 2:**
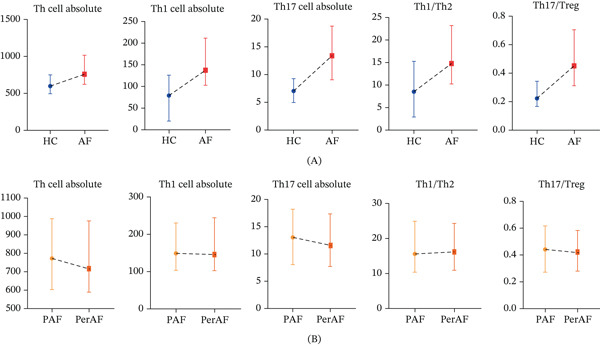
Comparison of absolute lymphocyte counts. (A) Lymphocyte counts between healthy controls (HC, blue) and patients with atrial fibrillation (AF, red). (B) Lymphocyte counts between patients with paroxysmal AF (PAF, light orange) and persistent AF (PerAF, dark orange). Data are presented as median and interquartile ranges. AF, atrial fibrillation; HC, healthy control; PAF, paroxysmal atrial fibrillation; PerAF, persistent atrial fibrillation.

PSM yielded 86 well‐matched pairs of AF patients and healthy controls for subsequent analysis. Prior to PSM, the groups differed significantly in age and sex, but these differences were effectively balanced after matching (Table 1). Compared to matched healthy controls, AF patients exhibited significantly higher absolute counts of Th1, Th17, and Th cells (referring to total CD4^+^T helper cells, including Th1, Th2, Th17, Treg, and other subsets), as well as higher Th1/Th2 and Th17/Treg ratios (*p*_FDR_ < 0.05) (Table 2, Figure 2A).

**Table 1:** Comparison of demographic characteristics before and after propensity score matching between healthy control and AF groups.

	Before propensity score matching			After propensity score matching		
Variables	Healthy control (*n* = 183)	AF patients (*n* = 302)	*p* value	Healthy control (*n* = 86)	AF patients (*n* = 86)	*p* value
Age, years	38(30, 50)	64(56, 70)	**< 0.001** ^∗^	51(45, 58.8)	52(46, 64)	0.112
Sex, male, *n*%	44 (24%)	185 (61.3%)	**< 0.001** ^∗^	28 (32.6%)	39 (45.3%)	0.085

*Note:* Boldface *p* values indicate significant differences before propensity score matching (*p* < 0.05).

Abbreviation: AF: atrial fibrillation.

^∗^Significant difference.

**Table 2:** Baseline characteristics and absolute lymphocyte counts in AF and healthy controls.

Variables	Total number (*n* = 172)	Healthy control (*n* = 86)	AF patients (*n* = 86)	*p* value	*p*_*FDR*_
Age, years	52.0 (45.0, 60.0)	51.0 (45.0, 58.8)	52.0 (46.0, 64.0)	0.112	0.149
Sex, *n* (%)				0.085	0.145
Female	105 (61.0)	58 (67.4)	47 (54.7)		
Male	67 (39.0)	28 (32.6)	39 (45.3)		
Hypertension, *n* (%)				**< 0.001** ^∗^	**< 0.001** ^∗^
No	134 (77.9)	86 (100)	48 (55.8)		
Yes	38 (22.1)	0 (0)	38 (44.2)		
Diabetes, *n* (%)				**0.003** ^∗^	**0.00638**
No	163 (94.8)	86 (100)	77 (89.5)		
Yes	9 (5.2)	0 (0)	9 (10.5)		
CHD, *n* (%)				**< 0.001** ^∗^	**< 0.001** ^∗^
No	156 (90.7)	86 (100)	70 (81.4)		
Yes	16 (9.3)	0 (0)	16 (18.6)		
Th1 cells/*μ*L	111.5 (64.6, 173.0)	78.8 (21.5, 122.5)	137.8 (102.9, 209.9)	**< 0.001** ^∗^	**< 0.001** ^∗^
Th2 cells/*μ*L	9.2 (5.8, 13.1)	8.9 (5.0, 12.7)	9.6 (6.5, 13.1)	0.114	0.149
Th17 cells/*μ*L	9.2 (6.1, 14.8)	7.0 (5.0, 9.2)	13.4 (9.3, 18.6)	**< 0.001** ^∗^	**< 0.001** ^∗^
Treg cells/*μ*L	31.0 (22.9, 39.0)	31.0 (22.1, 41.4)	30.6 (23.3, 37.2)	0.72	0.7664
Th1/Th2	11.8 (7.3, 18.5)	8.6 (2.9, 15.3)	14.8 (10.3, 23.2)	**< 0.001** ^∗^	**< 0.001** ^∗^
Th17/Treg	0.3 (0.2, 0.5)	0.2 (0.2, 0.3)	0.5 (0.3, 0.7)	**< 0.001** ^∗^	**< 0.001** ^∗^
T cells/*μ*L	1295.0 ± 415.2	1250.8 ± 378.2	1339.2 ± 447.0	0.163	0.198
B cells/*μ*L	209.6 (143.1, 279.9)	185.0 (137.2, 271.0)	223.6 (164.1, 305.4)	**0.04** ^∗^	0.077
Th cells/*μ*L^a^	685.6 (533.5, 922.7)	599.9 (501.3, 748.4)	760.1(628.6, 1019.0)	**< 0.001** ^∗^	**< 0.001** ^∗^
Ts cells/*μ*L	442.8 (317.8, 642.5)	447.0 (323.8, 651.8)	442.1 (315.7, 600.7)	0.806	0.806
Th/Ts	1.6 (1.3, 2.4)	1.8 (1.4, 2.3)	1.6 (1.2, 2.4)	0.636	0.721
NK cells/*μ*L	254.9 (173.8, 384.2)	278.6 (180.3, 424.8)	231.6 (170.4, 340.7)	0.114	0.149

*Note:* Boldface *p* values and *p*_FDR_ values indicate statistically significant differences between healthy controls and AF patients.

Abbreviations: AF: atrial fibrillation; CHD: coronary atherosclerotic heart disease; IQR: interquartile range.

^a^Th cells refer to total CD4^+^T helper cells (including Th1, Th2, Th17, Treg, and other subsets).

^∗^Significant difference.

#### 3.1.2. Multivariable logistic regression analysis of absolute lymphocyte count and AF

Multivariable logistic regression, adjusted for age, sex, hypertension, diabetes, and CHD (all VIF<5, Table S7), identified several lymphocyte subsets as independent risk factors for AF (Table S7). These included absolute counts of Th1 cells (OR = 1.01, 95% CI: 1.01–1.02, *p* < 0.001), Th17 cells (OR = 1.22, 95% CI: 1.12–1.32, *p* < 0.001), Th cells (OR = 1.0, 95% CI: 1.000–1.001, *p* = 0.004), and B cells (OR = 1.01, 95% CI: 1.001–1.010, *p* = 0.022), as well as higher Th1/Th2 (OR = 1.04, 95% CI: 1.01–1.08, *p* = 0.011) and Th17/Treg ratios (OR = 1.63 per 0.1‐unit increase, 95% CI: 1.3–2.05, *p* < 0.001) (Table 3).

**Table 3:** Multivariate logistic regression analysis of absolute lymphocyte count and AF.

Variables	OR (95% *CI*)	*p* value
Th1 cells/*μ*L	**1.01 (1.01~1.02)**	**< 0.001** ^∗^
Th2 cells/*μ*L	1.05 (0.99~1.13)	0.111
Th17 cells/*μ*L	**1.22 (1.12~1.32)**	**< 0.001** ^∗^
Treg cells/*μ*L	0.99 (0.96~1.02)	0.342
Th1/Th2	**1.04 (1.01~1.08)**	**0.011** ^∗^
Th17/Treg ^∗^0.1	**1.63 (1.3~2.05)**	**< 0.001** ^∗^
T cells/*μ*L	1 (1~1.001)	0.326
B cells/*μ*L	**1.01 (1.001~1.010)**	**0.022** ^∗^
Th cells/*μ*L^a^	**1 (1~1.001)**	**0.004** ^∗^
Ts cells/*μ*L	1 (1.001~1.004)	0.478
Th/Ts	1.06 (0.72~1.56)	0.778
NK cells/*μ*L	1 (0.997~1.002)	0.697

*Note:* Adjustment factors include age, gender, hypertension, diabetes, and coronary atherosclerotic heart disease. Boldface values indicate statistically significant variables (*p* < 0.05) in the multivariate logistic regression analysis.

^a^Th cells refer to total CD4^+^ T helper cells (including Th1, Th2, Th17, Treg, and other subsets).

^∗^Significant difference.

#### 3.1.3. Baseline Data of Patients With Different Types of AF

Table S5 shows baseline characteristics of patients with paroxysmal AF and persistent AF. Significant differences (*p*_FDR_ < 0.05) were found in sex, hypertension, CHA2DS2‐VASc score, drinking, presence of thrombosis, LVEF, left atrial size, and left atrial enlargement between the two groups. Table S6 shows substantial differences in laboratory tests between the two groups, including blood urea nitrogen (BUN), creatinine (Cr), uric acid (UA), homocysteine (HCY), total bilirubin (TBIL), direct bilirubin (DBIL), indirect bilirubin (IBIL), chloride (Cl), prothrombin time (PT), and international normalized ratio (INR) (*p*_FDR_ < 0.05). Table S8 shows that the absolute counts of various types of lymphocytes were not significantly different between the two groups (*p*_FDR_ < 0.05). Figure 2B demonstrates the comparison of absolute lymphocyte counts between the different types of AF groups.

#### 3.1.4. Multivariable logistic regression analysis of absolute lymphocyte count and different types of AF

Multivariable logistic regression analysis was employed to control for clear confounders on different types of AF post univariate analysis for all statistically different variables. The final adjusted model included age, sex, hypertension, diabetes, CHD, smoking, alcohol use, BMI, Cr, DBIL, Cl, left atrial size, and LVEF. All variables demonstrated acceptable multicollinearity (VIF<5) (Table S9). No absolute lymphocyte count type was found to be a risk factor for predicting AF type (Table S10). Our findings suggested that absolute peripheral blood lymphocyte counts, particularly T helper cells, may be linked to AF onset but not progression between types of AF. These results suggested that lymphocyte and T lymphocyte subsets in AF patients are disturbed early in the disease and may induce AF.

### 3.2. MR studies

#### 3.2.1. Causal relationship between immune cells and AF

**Figure 3:**
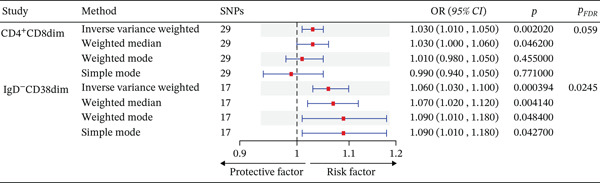
Forest plot of MR analysis results for two immune cells and AF. AF, atrial fibrillation; MR, Mendelian randomization.

We selected CD4^+^CD8dim T cell counts (T cells with low‐intensity expression of the CD8 marker) and IgD^−^CD38dim B cell counts (B cells with low‐intensity expression of IgD and CD38) for further analysis based on their significant associations with AF (Table S11). Genetically predicted CD4^+^CD8dim T cell counts were associated with an increased risk of AF (IVW: OR = 1.03, 95% CI: 1.01–1.05, *p* = 0.002, *p*_FDR_ = 0.059), supported by the WM method (OR = 1.03, *p* = 0.046). The mode‐based estimators were nonsignificant (weighted mode: OR = 1.01, 95% CI: 0.98–1.05, *p* = 0.455; simple mode: OR = 0.99, 95% CI: 0.94–1.05, *p* = 0.771), likely due to the limited number of SNPs (n = 29) and the lower power of mode‐based methods (Table S12 and Figure 3). Additionally, given the important role of B cells in the immune system, we included IgD^−^CD38dim B cell counts, which showed the strongest association with AF among B cell features (Table S12 and Figure 3). Genetically predicted IgD^−^CD38dim B cell counts were robustly associated with AF risk (IVW: OR = 1.06, 95% CI: 1.03–1.10, *p* = 0.0003, *p*_FDR_ = 0.0245), with consistent results from the WM (OR = 1.07, 95% CI: 1.02–1.12, *p* = 0.004), weighted mode (OR = 1.09, 95% CI: 1.01–1.18, *p* = 0.048), and simple mode (OR = 1.09, 95% CI: 1.01–1.18, *p* = 0.043) (Table S12 and Figure 3).

#### 3.2.2. Sensitivity analysis

Sensitivity analyses confirmed the robustness of the causal estimates. No significant heterogeneity was detected by Cochran′s *Q* test (*p* > 0.05). The MR‐Egger intercept test revealed no evidence of horizontal pleiotropy. MR‐PRESSO analysis indicated that causal estimates remained consistent after outlier removal. LOO analysis demonstrated that no single SNP drove the causal associations (Table S16 and Figures S3 and S4).

### 3.3. Drug‐Targeted MR Study

#### 3.3.1. Causal Relationship Between Target of Immunomodulatory Drugs and AF

##### 3.3.1.1. Two‐Sample MR Based on Publicly Available Genome‐Wide Association Studies (GWASs)

**Figure 4:**
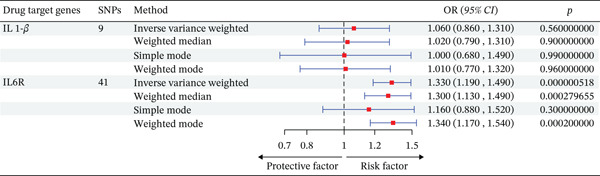
Forest plot of MR analysis of immunomodulatory drug target genes and AF. AF, atrial fibrillation; MR, Mendelian randomization.

Following the stringent filtering process described above, only IL‐1B and IL‐6R had SNPs available for further analysis. Nine and 41 SNPs were identified to represent the effects of IL‐1*β* and IL‐6R, respectively (Table S13). IL‐6R‐mediated CRP elevation was significantly associated with increased AF risk (IVW: OR = 1.33, 95% CI: 1.19–1.49, *p* = 5.18E‐7), supported by the WM (OR = 1.30, 95% CI: 1.13–1.49, *p* = 2.80E‐4) and weighted mode (OR = 1.34, 95% CI: 1.17–1.54, *p* = 2.00E‐4). The simple mode estimate was directionally consistent but nonsignificant (OR = 1.16, 95% CI: 0.88–1.52, *p* = 0.30). In contrast, no causal relationship was observed between IL‐1*β*‐mediated CRP elevation and AF risk (Table S14 and Figure 4).

##### 3.3.1.2. Two‐Sample MR Based on Expression Quantitative Trait Loci (eQTLs) Studies

**Figure 5:**
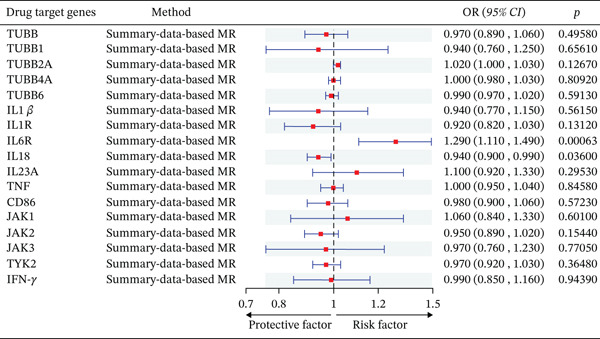
Forest plot of results of MR analysis of immunomodulatory drugs versus AF drug targets. AF, atrial fibrillation; MR, Mendelian randomization.

SMR analysis showed that IL‐6R gene expression in blood was associated with a higher risk of AF (OR = 1.29, 95% CI: 1.11–1.49, *p* = 6.3E‐4), suggesting that IL‐6R inhibitors may reduce AF risk (Table S17 and Figure 5). The expression of IL‐18 gene in blood was associated with lower incidence of AF (OR = 0.94, 95% CI: 0.90–0.99, *p* = 0.036), suggesting that the use of IL‐18 inhibitors may lead to the development of AF (Table S17 and Figure 5).

#### 3.3.2. Sensitivity analysis

##### 3.3.2.1. IVW‐MR Sensitivity Analysis

Cochran ‘s Q test found evidence of heterogeneity in IL‐6R results (*p* = 9.49E‐5) (Table S16). This heterogeneity may be explained by the fact that the two‐sample MR analysis used exposure and outcome data from distinct samples. The MR‐Egger intercept did not find evidence of pleiotropy (Table S16). LOO analysis further suggested that no SNPs alone could influence causal estimates in our analysis (Figure S5). Positive controls showed a substantial causal connection between IL‐6R‐mediated CRP rise and rheumatoid arthritis (OR = 1.34, 95% CI: 1.14–1.57, *p* = 4.12E‐4), confirming the study′s drug‐targeted gene IV selection (Table S15).

##### 3.3.2.2. SMR Sensitivity Analysis

For the SMR analysis, the HEIDI test showed no pleiotropy between IL‐18 expression and outcome (*p*_HEIDI_ = 0.79). In contrast, the HEIDI test showed pleiotropy between IL‐6R expression and outcome (*p*_HEIDI_ = 1.92E‐4) (Table S17). Gene clustering may link gene expression to outcome. Therefore, we identified three additional genes in the vicinity of IL‐6R (within a 1‐Mb window), screened them for eQTLs available in the gene‐wide level, and performed SMR analysis. The results showed that the expression of two genes was also associated with the occurrence of AF, which were the NUP210L gene and the SHE gene (Table S18).

## 4. Discussions

Immune cells such as macrophages, neutrophils, and lymphocytes are implicated in AF pathogenesis. Recent studies have shown that recruited SPP1+ macrophages interact with local immune and stromal cells to promote AF [29]. Additionally, acute inflammation, characterized by neutrophil predominance, has been observed in AF patients undergoing pericardiotomy, atriotomy, or catheter ablation [30].

Among lymphocytes, T cells may contribute to AF, though their precise mechanisms remain unclear. T lymphocytes are broadly categorized into Th cells (CD4^+^) and Ts cells (CD8^+^), with CD4^+^T cells further divided into subtypes such as Th1, Th2, Th17, and Treg. A large prospective cohort study by James [9] found that circulating immune cell subpopulations predicted new‐onset AF. Our CCS found CD4^+^cells as a risk factor for AF, suggesting that peripheral blood helper T‐lymphocyte imbalances may induce AF. MR analysis further implicated CD4^+^CD8dim T cells in AF risk.

In our study, elevated Th1 cells independently predicted AF risk. Th1 cells secrete inflammatory cytokines as interferon‐*γ* (IFN‐*γ*), IL‐2, and tumor necrosis factor‐*α* (TNF‐*α*), leading to inflammation and tissue damage [31]. Several bioinformatics studies using immune infiltration analysis have indicated that AF patients’ cardiac tissues have higher Th cell and Th1 cell counts than healthy individuals [32, 33]. Notably, inflammatory cytokines (e.g., IL‐6) and chemokines (e.g., SDF‐1*α*) upregulated in AF patients may further amplify Th1‐mediated inflammation, as demonstrated by increased mobilization of CD34^+^ HPCs in persistent AF, which exhibit a propensity to differentiate into cells expressing cardiomyocyte markers [34]. The role of IFN‐*γ* in fibrosis is context‐dependent. It may exert antifibrotic effects in myocardial infarction [8], but promotes fibroblast activation and extracellular matrix remodeling in pressure overload‐induced heart failure [35]. By contrast, Th2 cells secrete IL‐4 and IL‐13 to drive collagen deposition and fibrosis [8]. Our CCS revealed elevated Th1/Th2 ratios in AF patients. While the net effect of Th1 activation on atrial fibrosis remains unclear given the context‐dependent role of IFN‐*γ*, persistent Th1‐driven inflammation may disrupt the balance between pro‐ and antifibrotic signals in the atria, thereby contributing to atrial structural remodeling. Future studies incorporating direct fibrosis biomarkers or cardiac MRI are needed to validate whether Th1‐derived IFN‐*γ* drives atrial interstitial fibrosis in AF.

Th17 cells mainly generate IL‐17A/F, driving neutrophil recruitment and activation. A matched CCS examined the association between AF and Th17 cell‐related cytokines such as IL‐17A, IL‐17F, IL‐21, and IL‐22. High plasma levels of these cytokines independently increase AF risk. A basic study found that Th17 cell upregulation and Treg downregulation caused myocardial fibrosis and heart failure by increasing LOX expression [36], which could lead to matrix remodeling in AF. Treg cells, a CD4^+^T cell fraction with immunomodulatory effects, may maintain peripheral tolerance and avoid inflammatory diseases.

As previously mentioned, advanced age is the greatest risk factor for AF [4], and aging is closely linked to AF development. Our immune system, especially the lymphocyte‐based adaptive immune system, grows older with the accompanying signs of aging. Recent studies have revealed that intrinsic aging alterations in CD4^+^T cells cause chronic inflammation, which promotes body‐wide aging [37] Mittelbrunn [38] highlighted the top ten hallmarks of T cell dysfunction with aging. We suggest that CD4^+^CD8dim T cells, which are closely associated with AF development, may represent a chronically activated memory‐effector subset. These cells express the CD8*αα* homodimer, lack acute activation markers CD69 and CD25, yet exhibit enhanced Th1 cytokine production and cytotoxic potential [39, 40]. Such CD4^+^CD8dim T cells have been implicated in sustained inflammation in RA synovium [41] and chronic viral infections [39]. Whether these cells directly infiltrate atrial tissue and contribute to atrial inflammation in AF remains unknown. Given their proinflammatory and cytotoxic properties, CD4^+^CD8dim T cells may also be involved in the immunopathogenesis of AF, but direct evidence linking this subset to atrial inflammation is currently lacking. Future studies are needed to determine whether these cells infiltrate atrial tissue and actively contribute to inflammation and fibrosis in AF.

MR assumes that genetic variants influence the outcome exclusively through the exposure of interest. PhenoScanner V2 screening and complementary sensitivity analyses detected no evidence of directional pleiotropy for CD4^+^CD8dim T cells. However, GWAS summary statistics inherently reflect cell abundance rather than functional activation states, precluding direct inference of activation marker expression. Whether the observed associations represent cell subset–specific effects or reflect broader immunological mechanisms remains to be clarified. Future phenotypic studies incorporating activation and senescence markers, alongside tissue‐specific eQTL and colocalization analyses, are warranted to distinguish these possibilities.

Although there are no systematic standardized clinical trials of immune system modulation to treat AF, the prospects are promising. Colchicine, a cheap, widely used anti‐inflammatory, was originally proposed for the prevention of postoperative AF [42]. However, following clinical study found no statistically significant difference in the recurrence rate of AF in the colchicine group compared with the control group [43, 44]. Colchicine also failed to demonstrate an effect that could reduce the occurrence of AF in the MR analysis of this study, therefore high‐quality, large‐scale clinical trials are needed to confirm its efficacy. In addition, inflammatory cytokine–targeted therapies, like IL‐17A inhibitors [45] and IL‐1*β* inhibitors [46], may reduce AF incidence, but further clinical trials are needed to confirm efficacy. In the future, immune cell‐targeted treatments such macrophage reduction to reduce AF substrate susceptibility and tailored CD8^+^T cell therapy to alleviate cardiac fibrosis [47] may prevent and treat AF.

IL‐6R inhibition and high IL‐18 gene expression lower AF risk in the drug‐targeted MR research, but high IL‐6R expression increases it. IL‐6R exists in both soluble and membrane‐bound forms and blocks the proinflammatory properties of IL‐6 by blocking the signaling of soluble IL‐6R [48]. Thus, targeting IL‐6R has been shown to treat inflammatory disorders. Inflammation is linked to AF [49], and Kazuo [50] identified 35 novel AF susceptibility loci by cross‐ancestral genome analysis and IL‐6R as a putative causative gene by transcriptome association analysis, highlighting inflammatory signaling as a significant pathway and therapeutic target. However, our HEIDI test revealed potential pleiotropy for IL‐6R expression, and we found that AF was also associated with expression of the neighboring genes NUP210L and SHE (Table S18). Thus, further studies are needed to exclude genetic confounding before IL‐6R can be considered a definitive therapeutic target.

IL‐18 is a multifunctional cytokine involved in both innate and acquired immunity. Previous cohort studies have reported elevated IL‐18 levels in AF patients [51, 52], suggesting its role as a proinflammatory factor. However, our MR results showed that higher IL‐18 expression was associated with a lower risk of AF. Previous research indicated that chronic AF atrial tissue undergoes structural remodeling, without increased levels of proinflammatory cytokines such TNF‐*α* or IL‐1*β* [53]. This apparent paradox may be explained by the context‐dependent functions of IL‐18. In the presence of IL‐12, IL‐18 promotes Th1 responses; in the absence of IL‐12, it promotes Th2‐associated cytokine secretion [54]. Given the significant Th1/Th2 imbalance observed in our CCS, we speculate that elevated IL‐18 may represent a compensatory mechanism to restore immune balance. It remains unclear whether this protective effect reflects systemic or local actions, as circulating levels may not fully capture IL‐18 activity within atrial tissue. Feedback loops may also be involved; for instance, IL‐4 suppresses Th1 responses by downregulating IL‐18 receptor *α* chain expression on CD4^+^T cells [55], potentially creating a negative feedback loop that limits excessive inflammation. Notably, IL‐18 inhibitors have been used to treat autoimmune diseases including rheumatoid arthritis, which may raise the risk of AF in patients. Future studies measuring both circulating and tissue‐specific IL‐18 levels, as well as longitudinal assessments of IL‐18 expression before and after AF onset, are needed.

This study has several limitations. First, comorbidities were absent in healthy controls and thus could not be used for PSM, although multivariate models adjusted for hypertension, diabetes, and CHD. Unrecorded medications and heart failure status may represent residual confounders. Prospective studies with comprehensive medication and heart failure phenotyping are warranted. Second, no significant lymphocyte count differences were found between AF subtypes, possibly because the limited sample size masked small, clinically important variations. Larger cohorts are needed to explore lymphocyte counts in AF progression. Third, the immune cell GWAS data, whereas the most comprehensive available, are derived from a Sardinian genetic isolate, which may limit generalizability to broader European populations. We used PhenoScanner V2, which integrates multipopulation GWAS data, to exclude known pleiotropic SNPs. Future validation in independent European cohorts would further strengthen the evidence. Fourth, circulating IL‐18 expression served as a proxy for atrial tissue activity, and future atrial‐specific eQTL data are needed to localize the protective effect. Fifth, although this study reveals associations between T cell imbalances, genetic factors, and AF risk, these findings are observational and do not establish causation. Validation through large‐scale, multicenter trials with extended follow‐up and rigorous phenotyping is essential. MR‐derived therapeutic targets also require confirmation through controlled intervention trials to assess efficacy and safety.

## 5. Conclusions

Our study suggested that immunological dysfunction, particularly T lymphocyte subset abnormality, increases the incidence of AF. IL‐6R signaling may represent a potential target for AF prevention, although genetic confounding from neighboring genes requires further evaluation. IL‐18 inhibition could raise AF risk, with clinical trials necessary to validate causal effects.

## Author Contributions

Study design: Jiahui Li and Hongxuan Fan. Data analysis: Yafen Yang and Zhuolin Huang. Article drafting: Yalin Yuan and Rui Bai. Article revision: Jiahui Li and Xin Wang. Final approval: Bin Liang and Xin Wang.

## Funding

This study was supported by Research Project of Shanxi Provincial Health Commission (2018060).

## Disclosure

All authors contributed to the article and approved the submitted version. Jiahui Li, Yafen Yang and Hongxuan Fan have contributed to the work equally and should be regarded as cofirst authors.

## Ethics Statement

The study was approved by the ethics committee of the Second Hospital of Shanxi Medical University. ID (2022): YX No. 093.

## Conflicts of Interest

The authors declare that they have no conflict of interest.

## Supporting Information

Additional supporting information can be found online in the Supporting Information section.

## Supporting information


**Supporting Information 1** Table S1: GWAS information of 731 immune cell traits. Table S2: Information of eQTL and GWAS summary data. Table S3: Information of pleiotrophic IVs. Table S4: Targeted Gene Location and Ensembl ID of Multiple Immunomodulatory Drugs. Table S5: Comparison of baseline characteristics of different types of AF. Table S6: Comparison of laboratory parameters in different types of AF. Table S7: Collinearity test before multivariable logistic regression analysis of absolute lymphocyte counts and AF. Table S8: Absolute lymphocyte counts in different types of AF. Table S9: Collinearity test before multivariable logistic regression analysis of absolute lymphocyte count and different types of AF. Table S10: Multivariable logistic regression analysis of absolute lymphocyte count and different types of AF. Table S11: Significant MR results in immune cells and AF. Table S12: MR results of of CD4^+^CD8dim T and IgD^−^CD38dim B with AF. Table S13: Genetic variants used to proxy drug effects in MR primary analysis with AF as the outcome. Table S14: Mendelian randomization results of immunomodulatory drugs and AF. Table S15: MR results for the effect of the genetically proxied exposure on Rheumatoid arthritis. Table S16: Heterogeneity and pleiotropy tests of instrument effects. Table S17: SMR association between expression of gene and AF outcome. Table S18: Association between eQTL top SNP of IL6R with expression of other nearby genes.

**Supporting Information 2** Figure S1: MR Flow Chart. MR, Mendelian randomization. Figure S2: Flow Chart for Drug‐Targeted MR Studies. MR, Mendelian randomization. Figure S3: Sensitivity analysis of the causal relationship between CD4^+^CD8dim T cell count and AF. LOO analysis (A); MR‐Egger intercept test analysis (B). AF, atrial fibrillation. Figure S4: Sensitivity analysis of causal relationship between IgD^−^CD38dim B cell count and AF. LOO analysis (A); MR‐Egger intercept test analysis (B). AF, atrial fibrillation. Figure S5: Sensitivity analysis of causal relationship between IL‐6R and AF. LOO analysis (A); MR‐Egger intercept test analysis (B). AF, atrial fibrillation.

**Supporting Information 3** STROBE‐MR Checklist—A completed Strengthening the Reporting of Observational Studies in Epidemiology using Mendelian Randomization (STROBE‐MR) checklist is provided as a separate file.

## Data Availability

The data that support the findings of this study are available from the corresponding author upon reasonable request. It is noted that the genetic instruments used in this study were derived from publicly available GWAS summary data.
