# Supplementary material for: Analysis of the Correlation Between Helper T‐Lymphocyte Subsets and Atrial Fibrillation: An Observational and Mendelian Randomization Study
Source: Cardiovasc Ther. 2026 Aug 4;2026:4440768. doi: 10.1155/cdr/4440768 (PMC13438868; doi:10.1155/cdr/4440768)
Supplement: Supplementary file 1 [file CDR-2026-4440768-s001.docx]

- **FigureS1.** MR Flow Chart.

MR, Mendelian randomization.

-**FigureS2.** Flow Chart for Drug-Targeted MR Studies.

MR, Mendelian randomization.

- **FigureS3.**  Sensitivity analysis of the causal relationship between CD4^+^CD8dim T cell count and AF. LOO analysis (A); MR-Egger intercept test analysis (B).

AF, atrial fibrillation.

- **FigureS4.**  Sensitivity analysis of causal relationship between IgD^-^CD38dim B cell count and AF. LOO analysis (A); MR-Egger intercept test analysis (B).

AF, atrial fibrillation.

- **FigureS5.** Sensitivity analysis of causal relationship between IL-6R and AF. LOO analysis (A); MR-Egger intercept test analysis (B).

AF, atrial fibrillation.
